# (3*S*)-14,16-Dihy­droxy-3-methyl-3,4,5,6,9,10,11,12-octa­hydro-1*H*-2-benzoxacyclo­tetra­decine-1,7(8*H*)-dione (zearalanone) monohydrate

**DOI:** 10.1107/S1600536812018168

**Published:** 2012-04-28

**Authors:** Sarah Drzymala, Werner Kraus, Franziska Emmerling, Matthias Koch

**Affiliations:** a BAM Federal Institute for Materials Research and Testing, Department of Analytical Chemistry, Reference Materials, Richard-Willstätter-Strasse 11, D-12489 Berlin, Germany

## Abstract

The absolute configuration of the title compound, C_18_H_24_O_5_·H_2_O, was not been determined by anomalous-dispersion effects, but has been assigned by reference to an unchanging chiral centre in the synthetic procedure. Intra­molecular O—H⋯O hydrogen bonds stabilize the mol­ecular conformation. In the crystal, O—H⋯O hydrogen bonds link the main mol­ecules and the water mol­ecules, forming an infinite three-dimensional network.

## Related literature

For the preparation of zearalanone from natural zearalenone, see: Urry *et al.* (1966 ▶). For the crystal structures of zearalenone and its derivatives, see: Panneerselvam *et al.* (1996 ▶); Gelo-Pujić *et al.* (1994 ▶); Zhao *et al.* (2008 ▶). For the estrogenic and anabolic effects of zearalenone and its derivatives, see: Mirocha *et al.* (1968 ▶). For the exploitation of zearalanone as an inter­nal standard, see: Berthiller *et al.* (2005 ▶); Maragou *et al.* (2008 ▶); Ren *et al.* (2007 ▶); Shin *et al.* (2009 ▶).
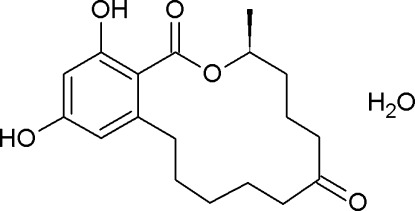


## Experimental

### 

#### Crystal data

C_18_H_24_O_5_·H_2_O
*M* *_r_* = 338.39
Orthorhombic, 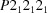

*a* = 8.2727 (11) Å
*b* = 24.579 (3) Å
*c* = 9.3703 (14) Å
*V* = 1905.3 (5) Å^3^
*Z* = 4
Mo *K*α radiation
μ = 0.09 mm^−1^
*T* = 296 K
0.45 × 0.25 × 0.1 mm

#### Data collection

Bruker APEXII CCD diffractometer
Absorption correction: multi-scan (*SADABS*; Bruker, 2001 ▶) *T*_min_ = 0.151, *T*_max_ = 0.477
20473 measured reflections
4626 independent reflections
2870 reflections with *I* > 2σ(*I*)
*R*_int_ = 0.109

#### Refinement

*R*[*F* ^2^ > 2σ(*F* ^2^)] = 0.051
*wR*(*F* ^2^) = 0.134
*S* = 0.90
4626 reflections
225 parameters
3 restraints
H atoms treated by a mixture of independent and constrained refinement
Δρ_max_ = 0.18 e Å^−3^
Δρ_min_ = −0.15 e Å^−3^

### 

Data collection: *APEX2* (Bruker, 2001 ▶); cell refinement: *SAINT* (Bruker, 2001 ▶); data reduction: *SAINT* (Bruker, 2001 ▶); program(s) used to solve structure: *SHELXS97* (Sheldrick, 2008 ▶); program(s) used to refine structure: *SHELXL97* (Sheldrick, 2008 ▶); molecular graphics: *SHELXTL* (Sheldrick, 2008 ▶); software used to prepare material for publication: *SHELXTL*.

## Supplementary Material

Crystal structure: contains datablock(s) I, global. DOI: 10.1107/S1600536812018168/fj2544sup1.cif

Structure factors: contains datablock(s) I. DOI: 10.1107/S1600536812018168/fj2544Isup2.hkl

Supplementary material file. DOI: 10.1107/S1600536812018168/fj2544Isup3.cml

Additional supplementary materials:  crystallographic information; 3D view; checkCIF report

## Figures and Tables

**Table 1:** Hydrogen-bond geometry (Å, °)

*D*—H⋯*A*	*D*—H	H⋯*A*	*D*⋯*A*	*D*—H⋯*A*
O4—H4*A*⋯O6	0.82	1.87	2.693 (2)	176
O5—H5*A*⋯O2	0.82	1.86	2.581 (2)	147
O6—H6*C*⋯O3^i^	0.96 (2)	1.85 (2)	2.810 (3)	178 (3)
O6—H6*D*⋯O5^ii^	0.96 (2)	1.95 (2)	2.887 (2)	164 (2)

Symmetry codes: (i) 
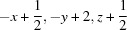
; (ii) 
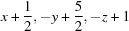
.

**Fig. 1.**
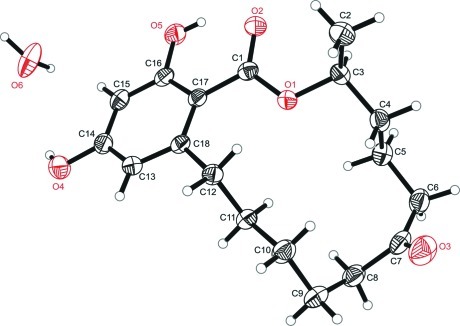
: ORTEP representation of the title compound with atomic labeling shown with 30% probability displacement ellipsoids.

**Fig. 2.**
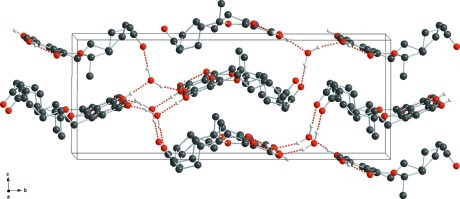
: View of the unit cell of the title compound, showing the hydrogen-bonded network. Hydrogen bonds are drawn as dashed red lines.

## supplementary crystallographic information

### Comment

Zearalanone (ZAN) is a semisynthetic resorcylic acid lactone (RAL) belonging to the group of Zearalenone (ZEN) analoga. ZAN was first prepared by catalytic hydrogenation of the double bond between C11 and C12 of natural ZEN (Urry *et al.*, 1966).

ZEN is a well known crop contaminant produced by a variety of *Fusarium* fungi. Its crystal structure was elucidated by Panneerselvam and colleagues (1996). Since the first isolation of ZEN from Fungi, a range of structurally closely related analoga have been isolated or prepared from ZEN (Gelo-Pujić *et al.*, 1994, Zhao *et al.*, 2008).

These RALs exhibit interesting estrogenic and anabolic effects due to their coupling with the estrogenic receptors alpha and beta (Mirocha *et al.*, 1968). Hence, ZAN was patented as a growth promoter in cattle as early as 1966 (U. S. P., 3239354). Furthermore, ZAN is not occurring in food, wherefore it was exploited as an internal standard for ZEN and its metabolites (Berthiller *et al.*, 2005, Maragou *et al.*, 2008, Ren *et al.*, 2007, Shin *et al.*, 2009).

The compound crystallizes in the orthorhombic space group *P*2_1_2_1_2_1_. The compound has a macrocyclic structure. The molecular structure of the compound and the atom-labeling scheme are shown in Fig 1. The absolute configuration could not be defined confidently based on the single-crystal diffraction data. The isomeric purity of the title compound was confirmed by ^1^H-NMR, HPLC-DAD and –MS/MS data. Besides the intramolecular hydrogen bonds between O5—H5A and O2, each molecule is connected to three adjacent water molecules *via* intermolecular hydrogen bonds (see dashed red bonds in Fig. 2). As a result a three dimensional network is formed.

### Experimental

Zearalanone was obtained from Toronto Research Chemicals (Canada, purity 98.0%). 5 mg (15.6 *µ*mol) were weighed in a 1.5 ml HPLC glass vial and solved in 0.5 ml DCM. Subsequently, 0.3 ml of *n*-Hexane were added. Colorless crystals of the title compound were formed after 7 days of slow solvent evaporation at room temperature.

### Refinement

All H-atoms were positioned geometrically and refined using a riding model with d(C—H) = 0.93 Å, *U*_iso_=1.2*U*_eq_ (C) for aromatic 0.98 Å, *U*_iso_ = 1.2*U*_eq_ (C) for CH, 0.97 Å, *U*_iso_ = 1.2*U*_eq_ (C) for CH_2_, 0.96 Å, *U*_iso_ = 1.5*U*_eq_ (C) for CH_3_ atoms, and 0.82 Å, *U*_iso_ = 1.5*U*_eq_ (C) for hydroxyl groups. The water hydrogen atoms were treated independently. In the absence of significant anomalous dispersion effects, Friedel pairs were merged.

### Crystal data

**Table:**

C_18_H_24_O_5_·H_2_O	*F*(000) = 728
*M**_r_* = 338.39	*D*_x_ = 1.180 Mg m^−^^3^
Orthorhombic, *P*2_1_2_1_2_1_	Mo *K*α radiation, λ = 0.71073 Å
Hall symbol: P 2ac 2ab	Cell parameters from 5584 reflections
*a* = 8.2727 (11) Å	θ = 2.3–25.7°
*b* = 24.579 (3) Å	µ = 0.09 mm^−^^1^
*c* = 9.3703 (14) Å	*T* = 296 K
*V* = 1905.3 (5) Å^3^	Block, colourless
*Z* = 4	0.45 × 0.25 × 0.1 mm

### Data collection

**Table:**

Bruker APEXII CCD diffractometer	4626 independent reflections
Radiation source: fine-focus sealed tube	2870 reflections with *I* > 2σ(*I*)
Graphite monochromator	*R*_int_ = 0.109
φ and ω scans	θ_max_ = 28.2°, θ_min_ = 1.7°
Absorption correction: multi-scan (*SADABS*; Bruker, 2001)	*h* = −10→10
*T*_min_ = 0.151, *T*_max_ = 0.477	*k* = −32→23
20473 measured reflections	*l* = −12→12

### Refinement

**Table:**

Refinement on *F*^2^	Primary atom site location: structure-invariant direct methods
Least-squares matrix: full	Secondary atom site location: difference Fourier map
*R*[*F*^2^ > 2σ(*F*^2^)] = 0.051	Hydrogen site location: inferred from neighbouring sites
*wR*(*F*^2^) = 0.134	H atoms treated by a mixture of independent and constrained refinement
*S* = 0.90	*w* = 1/[σ^2^(*F*_o_^2^) + (0.0708*P*)^2^] where *P* = (*F*_o_^2^ + 2*F*_c_^2^)/3
4626 reflections	(Δ/σ)_max_ = 0.001
225 parameters	Δρ_max_ = 0.18 e Å^−^^3^
3 restraints	Δρ_min_ = −0.15 e Å^−^^3^

### Special details

**Table:**

Geometry. All e.s.d.'s (except the e.s.d. in the dihedral angle between two l.s. planes) are estimated using the full covariance matrix. The cell e.s.d.'s are taken into account individually in the estimation of e.s.d.'s in distances, angles and torsion angles; correlations between e.s.d.'s in cell parameters are only used when they are defined by crystal symmetry. An approximate (isotropic) treatment of cell e.s.d.'s is used for estimating e.s.d.'s involving l.s. planes.
Refinement. Refinement of *F*^2^ against ALL reflections. The weighted *R*-factor *wR* and goodness of fit *S* are based on *F*^2^, conventional *R*-factors *R* are based on *F*, with *F* set to zero for negative *F*^2^. The threshold expression of *F*^2^ > σ(*F*^2^) is used only for calculating *R*-factors(gt) *etc*. and is not relevant to the choice of reflections for refinement. *R*-factors based on *F*^2^ are statistically about twice as large as those based on *F*, and *R*- factors based on ALL data will be even larger.

### Fractional atomic coordinates and isotropic or equivalent isotropic displacement parameters (Å^2^)

**Table:**

	*x*	*y*	*z*	*U*_iso_*/*U*_eq_	
O1	−0.02619 (16)	0.98111 (5)	0.36315 (17)	0.0478 (4)	
O2	−0.16664 (17)	1.05917 (5)	0.35375 (17)	0.0554 (4)	
O3	0.1136 (3)	0.77977 (7)	0.4127 (2)	0.0839 (6)	
O4	0.5417 (2)	1.15308 (6)	0.4798 (2)	0.0680 (5)	
H4A	0.5221	1.1805	0.5264	0.102*	
O5	−0.03447 (19)	1.14487 (6)	0.46197 (19)	0.0616 (4)	
H5A	−0.1084	1.1253	0.4345	0.092*	
C1	−0.0353 (2)	1.03555 (8)	0.3654 (2)	0.0425 (5)	
C2	−0.2267 (3)	0.95504 (10)	0.1831 (2)	0.0589 (6)	
H2A	−0.2494	0.9924	0.1610	0.088*	
H2B	−0.3218	0.9335	0.1667	0.088*	
H2C	−0.1406	0.9422	0.1232	0.088*	
C3	−0.1771 (2)	0.95029 (8)	0.3378 (2)	0.0455 (5)	
H3A	−0.2632	0.9647	0.3988	0.055*	
C4	−0.1378 (3)	0.89190 (8)	0.3826 (2)	0.0505 (5)	
H4B	−0.2274	0.8687	0.3553	0.061*	
H4C	−0.0435	0.8799	0.3299	0.061*	
C5	−0.1054 (3)	0.88393 (8)	0.5415 (2)	0.0552 (5)	
H5B	−0.2021	0.8935	0.5945	0.066*	
H5C	−0.0201	0.9086	0.5708	0.066*	
C6	−0.0556 (3)	0.82491 (9)	0.5808 (3)	0.0654 (7)	
H6A	−0.0491	0.8215	0.6837	0.078*	
H6B	−0.1377	0.7998	0.5470	0.078*	
C7	0.1051 (3)	0.80991 (8)	0.5160 (3)	0.0589 (6)	
C8	0.2570 (3)	0.83280 (9)	0.5834 (3)	0.0615 (6)	
H8A	0.2925	0.8081	0.6578	0.074*	
H8B	0.2305	0.8672	0.6284	0.074*	
C9	0.3968 (3)	0.84196 (8)	0.4802 (3)	0.0694 (7)	
H9A	0.4214	0.8079	0.4328	0.083*	
H9B	0.4916	0.8526	0.5344	0.083*	
C10	0.3630 (3)	0.88553 (8)	0.3663 (3)	0.0606 (6)	
H10A	0.4530	0.8861	0.3000	0.073*	
H10B	0.2675	0.8749	0.3132	0.073*	
C11	0.3374 (3)	0.94306 (7)	0.4230 (2)	0.0507 (5)	
H11A	0.4313	0.9536	0.4786	0.061*	
H11B	0.2447	0.9431	0.4863	0.061*	
C12	0.3099 (3)	0.98564 (7)	0.3038 (2)	0.0464 (5)	
H12A	0.4046	0.9868	0.2428	0.056*	
H12B	0.2187	0.9744	0.2458	0.056*	
C13	0.4138 (3)	1.07391 (8)	0.3932 (2)	0.0477 (5)	
H13A	0.5159	1.0599	0.3737	0.057*	
C14	0.4003 (3)	1.12590 (8)	0.4520 (2)	0.0510 (5)	
C15	0.2481 (3)	1.14844 (8)	0.4758 (2)	0.0516 (5)	
H15A	0.2387	1.1831	0.5144	0.062*	
C16	0.1105 (3)	1.11881 (8)	0.4414 (2)	0.0463 (5)	
C17	0.1200 (2)	1.06443 (7)	0.3887 (2)	0.0410 (5)	
C18	0.2776 (2)	1.04239 (7)	0.3628 (2)	0.0415 (4)	
O6	0.4912 (3)	1.24388 (7)	0.6343 (2)	0.1064 (8)	
H6C	0.458 (5)	1.2351 (13)	0.730 (2)	0.162 (17)*	
H6D	0.484 (5)	1.2781 (8)	0.585 (3)	0.142 (14)*	

### Atomic displacement parameters (Å^2^)

**Table:**

	*U*^11^	*U*^22^	*U*^33^	*U*^12^	*U*^13^	*U*^23^
O1	0.0409 (7)	0.0411 (7)	0.0614 (9)	0.0008 (6)	−0.0041 (7)	0.0008 (7)
O2	0.0449 (8)	0.0494 (7)	0.0720 (10)	0.0083 (6)	0.0012 (8)	0.0019 (7)
O3	0.1077 (16)	0.0612 (10)	0.0827 (13)	0.0028 (10)	−0.0138 (12)	−0.0180 (10)
O4	0.0628 (10)	0.0552 (9)	0.0859 (13)	−0.0091 (8)	−0.0132 (9)	−0.0058 (9)
O5	0.0568 (9)	0.0424 (7)	0.0856 (12)	0.0099 (7)	0.0087 (9)	−0.0011 (8)
C1	0.0454 (11)	0.0442 (10)	0.0379 (10)	0.0057 (9)	0.0052 (10)	0.0050 (9)
C2	0.0575 (14)	0.0706 (14)	0.0487 (12)	0.0015 (11)	−0.0035 (11)	−0.0040 (11)
C3	0.0379 (10)	0.0493 (10)	0.0493 (12)	−0.0020 (9)	0.0003 (10)	−0.0056 (9)
C4	0.0491 (13)	0.0473 (11)	0.0551 (13)	−0.0063 (9)	−0.0022 (11)	−0.0060 (9)
C5	0.0613 (14)	0.0511 (11)	0.0531 (13)	−0.0053 (11)	0.0037 (13)	0.0009 (10)
C6	0.0741 (16)	0.0544 (13)	0.0676 (16)	−0.0143 (12)	−0.0036 (14)	0.0111 (11)
C7	0.0837 (17)	0.0355 (9)	0.0574 (14)	−0.0020 (11)	−0.0090 (14)	0.0074 (10)
C8	0.0753 (16)	0.0467 (11)	0.0627 (15)	0.0016 (11)	−0.0158 (14)	0.0093 (11)
C9	0.0693 (16)	0.0412 (11)	0.098 (2)	0.0113 (11)	0.0005 (16)	0.0084 (12)
C10	0.0639 (15)	0.0454 (11)	0.0725 (16)	0.0075 (10)	0.0122 (14)	0.0020 (11)
C11	0.0510 (12)	0.0391 (10)	0.0622 (13)	0.0030 (9)	−0.0041 (11)	0.0017 (9)
C12	0.0448 (11)	0.0426 (10)	0.0517 (12)	0.0038 (9)	0.0053 (10)	−0.0010 (9)
C13	0.0441 (11)	0.0449 (10)	0.0541 (13)	0.0032 (9)	−0.0006 (10)	0.0044 (9)
C14	0.0553 (13)	0.0465 (10)	0.0511 (13)	−0.0085 (10)	−0.0081 (12)	0.0078 (10)
C15	0.0659 (14)	0.0341 (9)	0.0548 (13)	−0.0002 (10)	0.0007 (12)	−0.0004 (9)
C16	0.0535 (12)	0.0386 (9)	0.0469 (12)	0.0071 (9)	0.0053 (11)	0.0091 (9)
C17	0.0452 (11)	0.0381 (9)	0.0398 (11)	0.0024 (8)	0.0015 (10)	0.0070 (8)
C18	0.0476 (11)	0.0379 (9)	0.0391 (10)	0.0032 (8)	0.0003 (10)	0.0078 (8)
O6	0.187 (3)	0.0491 (10)	0.0833 (15)	−0.0237 (12)	0.0217 (17)	0.0017 (10)

### Geometric parameters (Å, º)

**Table:**

O1—C1	1.340 (2)	C8—C9	1.524 (4)
O1—C3	1.479 (2)	C8—H8A	0.9700
O2—C1	1.237 (2)	C8—H8B	0.9700
O3—C7	1.221 (3)	C9—C10	1.538 (3)
O4—C14	1.372 (3)	C9—H9A	0.9700
O4—H4A	0.8200	C9—H9B	0.9700
O5—C16	1.373 (3)	C10—C11	1.525 (3)
O5—H5A	0.8200	C10—H10A	0.9700
C1—C17	1.484 (3)	C10—H10B	0.9700
C2—C3	1.511 (3)	C11—C12	1.548 (3)
C2—H2A	0.9600	C11—H11A	0.9700
C2—H2B	0.9600	C11—H11B	0.9700
C2—H2C	0.9600	C12—C18	1.524 (3)
C3—C4	1.531 (3)	C12—H12A	0.9700
C3—H3A	0.9800	C12—H12B	0.9700
C4—C5	1.525 (3)	C13—C18	1.396 (3)
C4—H4B	0.9700	C13—C14	1.396 (3)
C4—H4C	0.9700	C13—H13A	0.9300
C5—C6	1.552 (3)	C14—C15	1.394 (3)
C5—H5B	0.9700	C15—C16	1.389 (3)
C5—H5C	0.9700	C15—H15A	0.9300
C6—C7	1.507 (4)	C16—C17	1.427 (3)
C6—H6A	0.9700	C17—C18	1.433 (3)
C6—H6B	0.9700	O6—H6C	0.960 (10)
C7—C8	1.515 (3)	O6—H6D	0.961 (10)
			
C1—O1—C3	117.78 (15)	H8A—C8—H8B	107.5
C14—O4—H4A	109.5	C8—C9—C10	113.91 (19)
C16—O5—H5A	109.5	C8—C9—H9A	108.8
O2—C1—O1	121.12 (18)	C10—C9—H9A	108.8
O2—C1—C17	123.31 (17)	C8—C9—H9B	108.8
O1—C1—C17	115.53 (16)	C10—C9—H9B	108.8
C3—C2—H2A	109.5	H9A—C9—H9B	107.7
C3—C2—H2B	109.5	C11—C10—C9	115.4 (2)
H2A—C2—H2B	109.5	C11—C10—H10A	108.4
C3—C2—H2C	109.5	C9—C10—H10A	108.4
H2A—C2—H2C	109.5	C11—C10—H10B	108.4
H2B—C2—H2C	109.5	C9—C10—H10B	108.4
O1—C3—C2	110.14 (18)	H10A—C10—H10B	107.5
O1—C3—C4	104.88 (15)	C10—C11—C12	113.32 (19)
C2—C3—C4	113.18 (18)	C10—C11—H11A	108.9
O1—C3—H3A	109.5	C12—C11—H11A	108.9
C2—C3—H3A	109.5	C10—C11—H11B	108.9
C4—C3—H3A	109.5	C12—C11—H11B	108.9
C5—C4—C3	115.21 (17)	H11A—C11—H11B	107.7
C5—C4—H4B	108.5	C18—C12—C11	112.51 (17)
C3—C4—H4B	108.5	C18—C12—H12A	109.1
C5—C4—H4C	108.5	C11—C12—H12A	109.1
C3—C4—H4C	108.5	C18—C12—H12B	109.1
H4B—C4—H4C	107.5	C11—C12—H12B	109.1
C4—C5—C6	113.45 (18)	H12A—C12—H12B	107.8
C4—C5—H5B	108.9	C18—C13—C14	121.60 (19)
C6—C5—H5B	108.9	C18—C13—H13A	119.2
C4—C5—H5C	108.9	C14—C13—H13A	119.2
C6—C5—H5C	108.9	O4—C14—C15	123.12 (18)
H5B—C5—H5C	107.7	O4—C14—C13	116.9 (2)
C7—C6—C5	111.56 (19)	C15—C14—C13	119.94 (19)
C7—C6—H6A	109.3	C16—C15—C14	119.67 (18)
C5—C6—H6A	109.3	C16—C15—H15A	120.2
C7—C6—H6B	109.3	C14—C15—H15A	120.2
C5—C6—H6B	109.3	O5—C16—C15	116.03 (17)
H6A—C6—H6B	108.0	O5—C16—C17	122.26 (19)
O3—C7—C6	121.3 (2)	C15—C16—C17	121.71 (19)
O3—C7—C8	120.5 (3)	C16—C17—C18	117.60 (18)
C6—C7—C8	118.2 (2)	C16—C17—C1	116.80 (17)
C7—C8—C9	114.8 (2)	C18—C17—C1	125.60 (16)
C7—C8—H8A	108.6	C13—C18—C17	119.32 (17)
C9—C8—H8A	108.6	C13—C18—C12	116.14 (17)
C7—C8—H8B	108.6	C17—C18—C12	124.54 (17)
C9—C8—H8B	108.6	H6C—O6—H6D	128.6 (19)

### Hydrogen-bond geometry (Å, º)

**Table:**

*D*—H···*A*	*D*—H	H···*A*	*D*···*A*	*D*—H···*A*
O4—H4*A*···O6	0.82	1.87	2.693 (2)	176
O5—H5*A*···O2	0.82	1.86	2.581 (2)	147
O6—H6*C*···O3^i^	0.96 (2)	1.85 (2)	2.810 (3)	178 (3)
O6—H6*D*···O5^ii^	0.96 (2)	1.95 (2)	2.887 (2)	164 (2)

Symmetry codes: (i) −*x*+1/2, −*y*+2, *z*+1/2; (ii) *x*+1/2, −*y*+5/2, −*z*+1.

## References

[bb1] Berthiller, F., Schuhmacher, R., Buttinger, G. & Krska, R. (2005). *J. Chromatogr. A*, **1062**, 209–216. 10.1016/j.chroma.2004.11.011 15679158

[bb2] Bruker (2001). *APEX2*, *SAINT* and *SADABS* Bruker AXS Inc., Madison, Wisconsin, USA.

[bb3] Gelo-Pujić, M., Antolić, S., Kojić-Prodić, B. & Šunjić, V. (1994). *Tetrahedron*, **50**, 13753–13764.

[bb4] Maragou, N. C., Rosenberg, E., Thomaidis, N. S. & Koupparis, M. A. (2008). *J. Chromatogr. A*, **1202**, 47–57. 10.1016/j.chroma.2008.06.042 18621378

[bb5] Mirocha, C. J., Christensen, C. M. & Nelson, G. H. (1968). *Cancer Res.* **28**, 2319–2322. 5749422

[bb6] Panneerselvam, K., Rudiño-Piñera, E. & Soriano-García, M. (1996). *Acta Cryst.* C**52**, 3095–3097.

[bb7] Ren, Y., Zhang, Y., Shao, S., Cai, Z., Feng, L., Pan, H. & Wang, Z. (2007). *J. Chromatogr. A*, **1143**, 48–64. 10.1016/j.chroma.2006.12.064 17234198

[bb8] Sheldrick, G. M. (2008). *Acta Cryst.* A**64**, 112–122. 10.1107/S0108767307043930 18156677

[bb9] Shin, B. S., Hong, S. H., Hwang, S. W., Kim, H. J., Lee, J. B., Yoon, H. S., Kim do, J. & Yoo, S. D. (2009). *Biomed. Chromatogr.* **23**, 1014–1021. 10.1002/bmc.1217 19347967

[bb10] Urry, W. H., Wehrmeister, H. L., Hodge, E. B. & Hidy, P. H. (1966). *Tetrahedron Lett.* **7**, 3109–3114.

[bb11] Zhao, L.-L., Gai, Y., Kobayashi, H., Hu, C.-Q. & Zhang, H.-P. (2008). *Acta Cryst.* E**64**, o999. 10.1107/S1600536808010258 PMC2961568 21202724

